# Supplementary material for: Computational Retinal Microvascular Biomarkers from an OCTA Image in Clinical Investigation
Source: Biomedicines. 2024 Apr 15;12(4):868. doi: 10.3390/biomedicines12040868 (PMC11048516; doi:10.3390/biomedicines12040868)
Supplement: Supplementary file 1 [file biomedicines-12-00868-s001.zip › biomedicines-2817181-supplementary.pdf]

Supplemental material

Supplement to: OCTA CRMBs in clinical investigation

Contents

1 Study design and sample demographics **2**

2 Quantifying computational retinal microvascular biomarkers **3**

2.1 Data acquisition 3

2.2 Data pre-processing 4

2.2.1 Digital vasculature map generation 4

2.2.2 Early treatment of diabetic retinopathy grid application 5

2.2.3 Foveal avascular zone demarcation 6

2.2.4 Delineation of retinal large vessels and separation of vessel segments 7

2.3 Calculation of computational retinal microvascular biomarkers 9

2.3.1 Retinal capillaries biomarkers 9

2.3.2 Retinal large vessels biomarkers 9

2.3.3 Biomarkers related to the foveal avascular zone 11

1 Study design and sample demographics

The study examined 43 eyes of 43 patients with resolved retinal vein occlusion (RVO) macular edema (ME) and 30 control subjects at the Department of Ophthalmology of Eye Hospital of China Academy of Chinese Medicine Sciences (Beijing, China) between January 2016 and December 2019.). The baseline characteristics of the patients were summarized in Table 1.

All the patients were diagnosed based on colour fundus examination and fluorescein angiography (FA) findings by two retinal specialists (Dr. Xie, and Dr. Lu). At their initial visit, a confirmed history of RVO with retinal haemorrhage and ME extending to the fovea was present in each enrolled patient. Ischemic type of RVO was defined when the non-perfused area was larger than 5-disc areas on FA in branch retinal vein occlusion (BRVO) patients, and when the non-perfused area was larger than 10-disc areas on FA in central retinal vein occlusion (CRVO) patients. All participants received a single intravitreal conbercept (Lumitin; Chengdu Kang Hong Biotech Co, Ltd, Sichuan, China) (0.5 mg/0.05 mL) injection for ME secondary to RVO.

At the initial visit and after the resolution of RVO ME, each patient underwent a comprehensive ophthalmologic examination, including best-corrected visual acuity (BCVA) measurement, slit-lamp biomicroscopy, colour fundus photography, and spectral domain optical coherence tomography angiography (OCTA) examination on the same day. BCVA was measured using a standard logarithmic visual acuity chart, 45° digital fundus photography (TRC-50LX, Topcon, Tokyo, Japan) was performed after pupil dilation, whereas OCTA examination was performed using RTVue XR Avanti (RTVue XR Avanti, AngioVue; Optovue, Inc., Fremont, CA, USA). FA was performed if necessary. The inclusion criteria were as follows: no current ME after anti-VEGF treatment (foveal thickness [FT] < 300 μm and no retinal cysts), OCTA images with good quality (signal strength index > 50). Exclusion criteria included patients with retinal arterial occlusion, previous retinal surgery, history of uveitis, ocular trauma or any previous or combined retinal diseases that could affect the explanation of OCTA measurements, such as severe diabetic retinopathy, age-related macular degeneration, glaucoma, retinitis pigmentosa and pathologic myopia (> 8 dioptres). Moreover, subjects with OCTA images of poor quality for evaluation due to poor fixation, low signal strength index (< 50) or significant media opacity were excluded in the study.

Table 1: Patient demographics and baseline characteristics

	RVO	Control	p-value	Adjusted p-value
Number of Patients and Eyes	43 (43 eyes)	30 (30 eyes)	-	-
Sex (men/women)	19/24	17/13	0.2940	0.3360
Study eye (right/left)	22/21	15/15	0.9221	0.9221
Age, years	55.42 ± 12.95	44.74 ± 15.37	0.0029	0.0077
Subtype (BRVO/CRVO)	25/18	-	-	-
Subtype (ischemic/non-ischemic)	11/32	-	-	-
Diabetes mellitus, yes/no	2/41	0/30	0.2310	0.3080
Hypertension, yes/no	15/28	0/30	0.0003	0.0012
Hyperlipidaemia, yes/no	6/37	0/30	0.0327	0.0523
Atherosclerosis, yes/no	11/32	1/29	0.0117	0.0234
Duration from the initial visit, months	2.00 ± 2.06	-	-	-
BCVA at initial visit	0.43 ± 0.29	0.93 ± 0.10	<0.001	<0.001
BCVA at inclusion	0.73 ± 0.28	-	-	-

Continuous variables were expressed as mean ± SD with p-values from independent t-tests (2 tails), whereas binary variables were expressed as counts with p-values from proportion tests. Significant at adjusted p-value < 0.05. Abbreviations: RVO, retinal vein occlusion; BRVO, branch retinal vein occlusion; CRVO, central retinal vein occlusion; BCVA, best-corrected visual acuity.

2 Quantifying computational retinal microvascular biomarkers

2.1 Data acquisition

The OCTA imaging data used in our study was obtained using the RTVue XR Avanti (Optovue, Fremont, CA) (Software Version 2017.1). We chose a 3 mm × 3 mm scan area that was centered on the fovea to quantify OCTA parameters for all study eyes. Correct centering of the rings was checked for each OCTA image. The 3 mm × 3 mm OCTA images were evaluated for the following features using the built-in software: FAZ area, parafoveal VD in both superficial capillary plexus (SCP) and deep capillary plexus (DCP). FAZ area was semi-automatically corrected by two fully trained retina specialists (Dr. Xie, and Dr. Lu). The capillary dropouts on both plexus were judged by two fully trained retina specialists (Dr. Xie, and Dr. Lu) independently, who were not informed of the visual acuity or any other information regarding the enrolled patient, and were graded from 0 to 8 according to previous study [1] (0 being no capillary dropout and 8 being extensive capillary dropout). The original electronic output files containing raw OCTA images (Figure 1) of both superficial capillary plexus (SCP) and deep capillary plexus (DCP) were exported and used in our analyses.

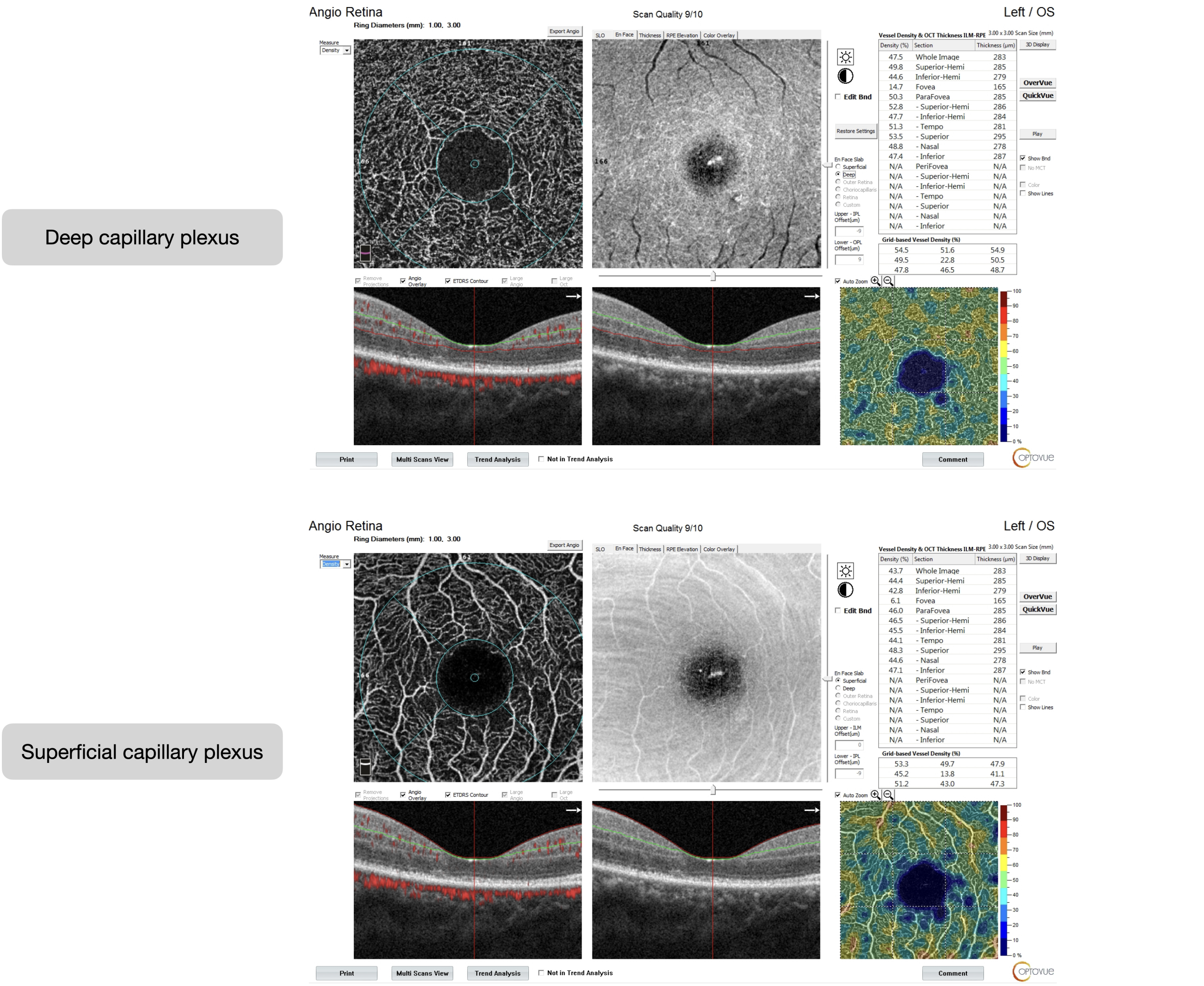

Figure 1: Example of raw Optovue output images in the superficial and deep capillary plexuses.

2.2 Data pre-processing

2.2.1 Digital vasculature map generation

The digital vasculature map (DVM) corresponding to the 3×3 scan area was generated from the original Optovue output file for subsequent analyses (Figure 2). The OCTA image was first converted to grayscale and thresholded using Otsu's binarization [2]. All the vertical/horizontal line segments in the resulting image were identified through Hough line transform [3] and the detected line segments were combined into a new image. The contours in the new image corresponded to rectangles in the original OCTA image. The top left rectangle with a width/height larger than $0.27 \times$ the width of the original OCTA image was next extracted. If the rectangle did not have an equal length and width, it was cropped to square with cropping coordinates determined by performing Harris corner detection [4] on the padded, Gaussian-blurred and thresholded version of it. This square was thus the extracted DVM from the calibrated OCTA image.

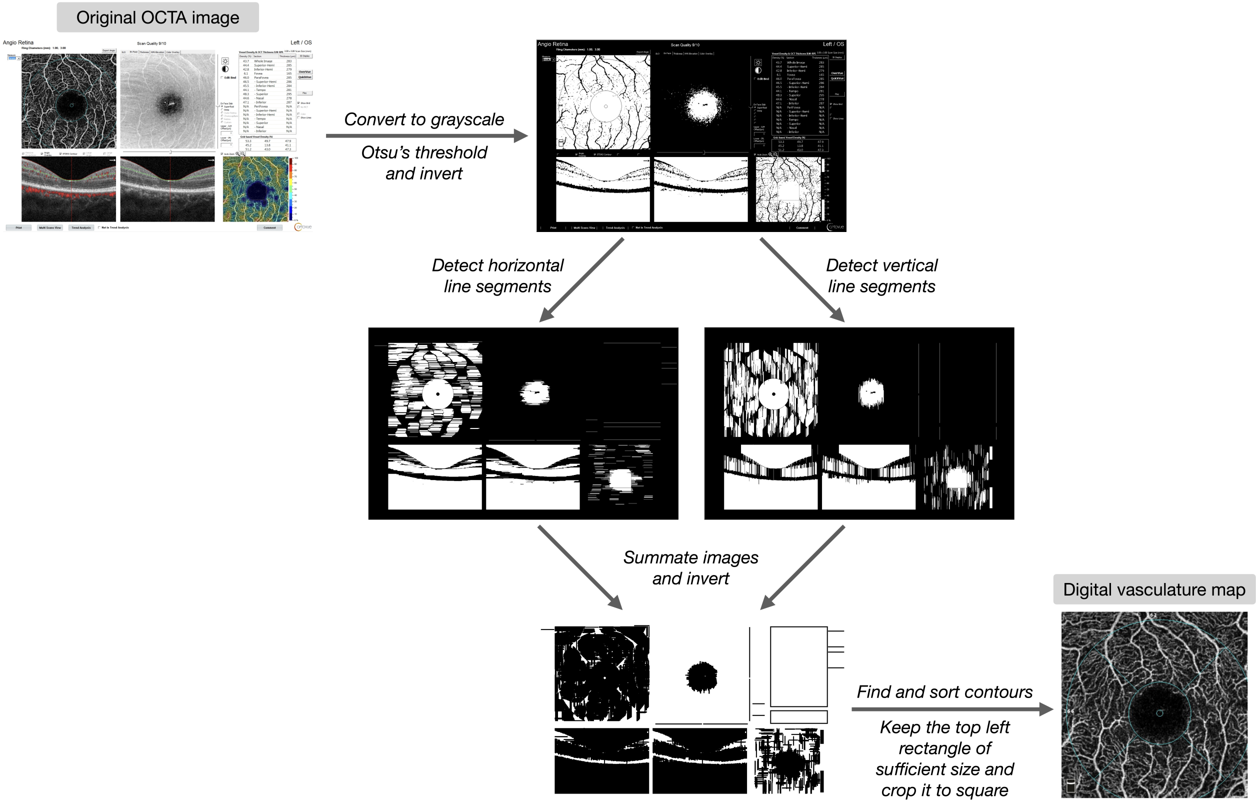

Figure 2: Process of digital vasculature map generation. Abbreviation used – OCTA: optical coherence tomography angiography.

2.2.2 Early treatment of diabetic retinopathy grid application

The early treatment of diabetic retinopathy (ETDRS) grid was next applied to the extracted DVM (Figure 3A). We utilized Optovue's reference circles to overlay the ETDRS grid properly. To drop most of the vessel signals, the map was converted to the International Commission on Illumination L*a*b* (Lab) color space [5] (L, a, b) and all the pixels with $abDist = \sqrt{a^2 + b^2} < 18$ were replaced with white ones to keep only the colored components. The resulting image was next converted to grayscale and skeletonized, and the reference circles in the ETDRS grid were identified through Hough circle transform [3] – the inner reference circle C_{inner} had the center (x_{inner}, y_{inner}) and a radius of r_{inner}, whereas the outer reference circle C_{outer} had the center $(x_{outer}, y_{outer}) = (x_{inner}, y_{inner})$ and a radius of r_{outer}. The area within C_{inner} roughly corresponded to the fovea region, whereas the parafovea area referred to the ring region between C_{inner} and C_{outer}.

Based on the ETDRS grid, we divided the parafovea into superior, inferior, nasal and tempo sections (Figure 3B) by calculating the section boundaries according to whether the studied eye is right (OD) or left (OS) using the recorded parameters of the reference circles. For instance, noting that the coordinates of the top left corner of a DVM are $(0, 0)$, the end points of the line segment dividing the superior and nasal sections of the right eye will be: $(x_{inner} + \frac{r_{inner}}{\sqrt{2}}, y_{inner} - \frac{r_{inner}}{\sqrt{2}})$ and $(x_{outer} + \frac{r_{outer}}{\sqrt{2}}, y_{outer} - \frac{r_{outer}}{\sqrt{2}})$.

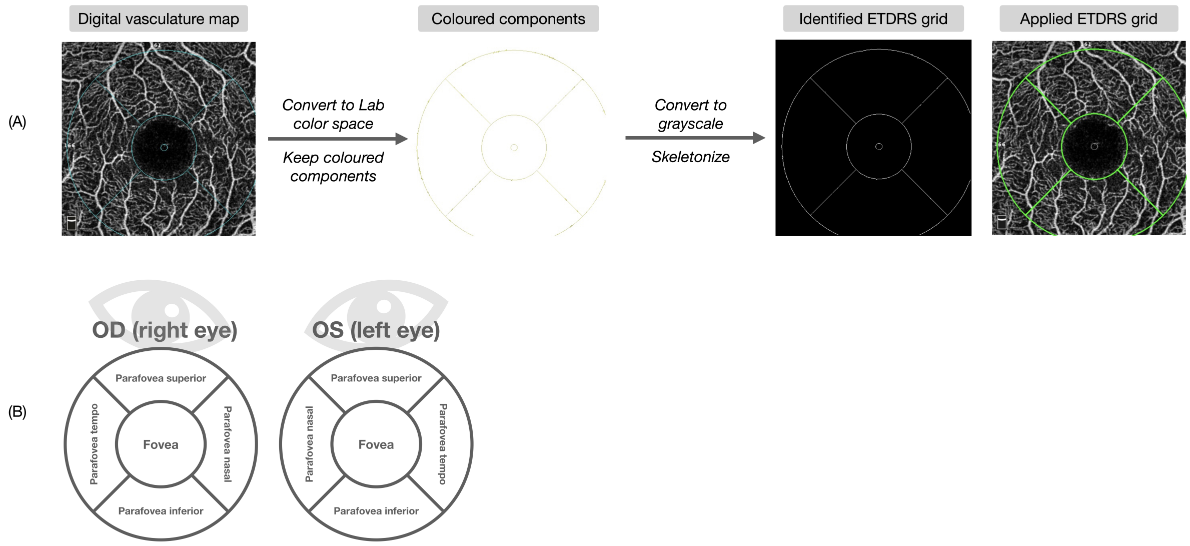

Figure 3: Application of the ETDRS grid. Abbreviation used – ETDRS: early treatment of diabetic retinopathy; Lab color space: International Commission on Illumination L*a*b* color space.

2.2.3 Foveal avascular zone demarcation

The foveal avascular zone (FAZ) is defined to be the capillary-free area within the innermost ring of the parafoveal capillary plexus. To demarcate the FAZ, we first masked the reference circles and lines representing the ETDRS grid in the DVM with a median filtered version of their corresponding areas in the map. Median filtering was next applied to the whole DVM to suppress noise. The masked and denoised image (Figure 4B) was adaptively thresholded and closed (dilation followed by erosion), followed by small objects removal. Subsequently, bridging (setting 0-valued pixels to 1 if they have two nonzero neighbors that are not connected) and thinning was applied to the resulting map (Figure 4C). Once again, small isolated objects were removed from the image to improve the robustness of the FAZ identification method.

The maximum polygon in the foveal avascular (i.e. the polygon does not cover any of the positive vessel signals) region of the resulting image (Figure 4D) was next obtained. Note that $(0, 0)$ corresponded to the top left corner of a DVM. Denote the center of the fovea region as (x_{inner}, y_{inner}) and the side length of the DVM as s. For $\theta = 0, 3, 6, 9, \ldots, 357$ (i.e. altogether 120 θ's), all the pixels within the image on the directed line segment from (x_{inner}, y_{inner}) to $(x_{inner} + s \cdot \sin\theta, y_{inner} + s \cdot \cos\theta)$ were considered. The first pixel with a positive signal (i.e. is part of a vessel) was recorded as one of the endpoints of the FAZ (half of these endpoints were shown as red dots in Figure 4E). If the values of all the pixels within the image on the directed line segment were zero, this particular θ was skipped. If less than three searches returned a positive signal, an error was reported. The polygon defined by connecting all the $n = 120$ (or less, if some of the searches did not find any positive pixels) endpoints in the order they were found would be the approximated FAZ (Figure 4F).

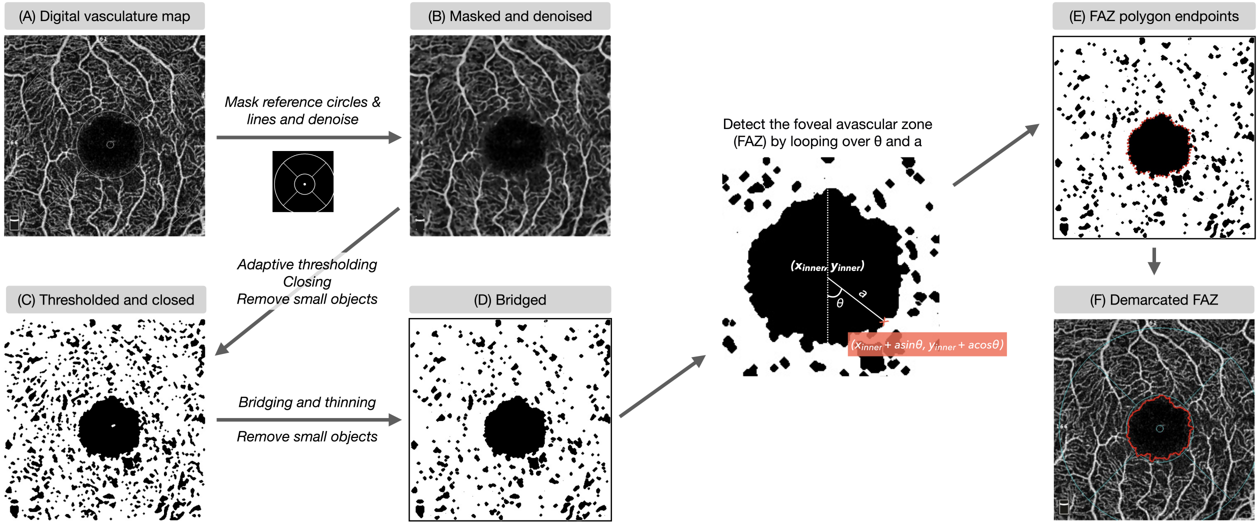

Figure 4: Process of foveal avascular zone demarcation.

2.2.4 Delineation of retinal large vessels and separation of vessel segments

In our study, we delineated the retinal large vessels in the SCP. This process is illustrated in Figure 5A to Figure 5G. The original vasculature map (Figure 5A) was first binarized through adaptive global thresholding, resulting in Figure 5B. Only the fovea and parafovea regions were retained according to the ETDRS grid. Next, Sato's filter [6] was utilized to detect continuous ridges, or the vessels, in the resulting vasculature map (Figure 5C). The ridge-filtered image (Figure 5D) was again binarized through adaptive thresholding based on the median of parafoveal pixel intensity, and small objects, which may be insignificant isolated vessel branches or noise, were removed from the binarized image (Figure 5E) for better robustness. Finally, all the contours in the resulting map (Figure 5F) were identified. Each contour corresponded to one delineated retinal vessel, as shown in Figure 5G.

Next, we separated the retinal vessel segments (Figure 5F to Figure 5I). Firstly, the vasculature map with delineated retinal vessels (Figure 5F) was skeletonized. The endpoints and branch points in the skeletonized image (Figure 5H) were detected through the algorithm shown in Figure 6. The identified branch points were next masked from the image and all the contours in the masked image were found. Each identified contour corresponded to one vessel segment.

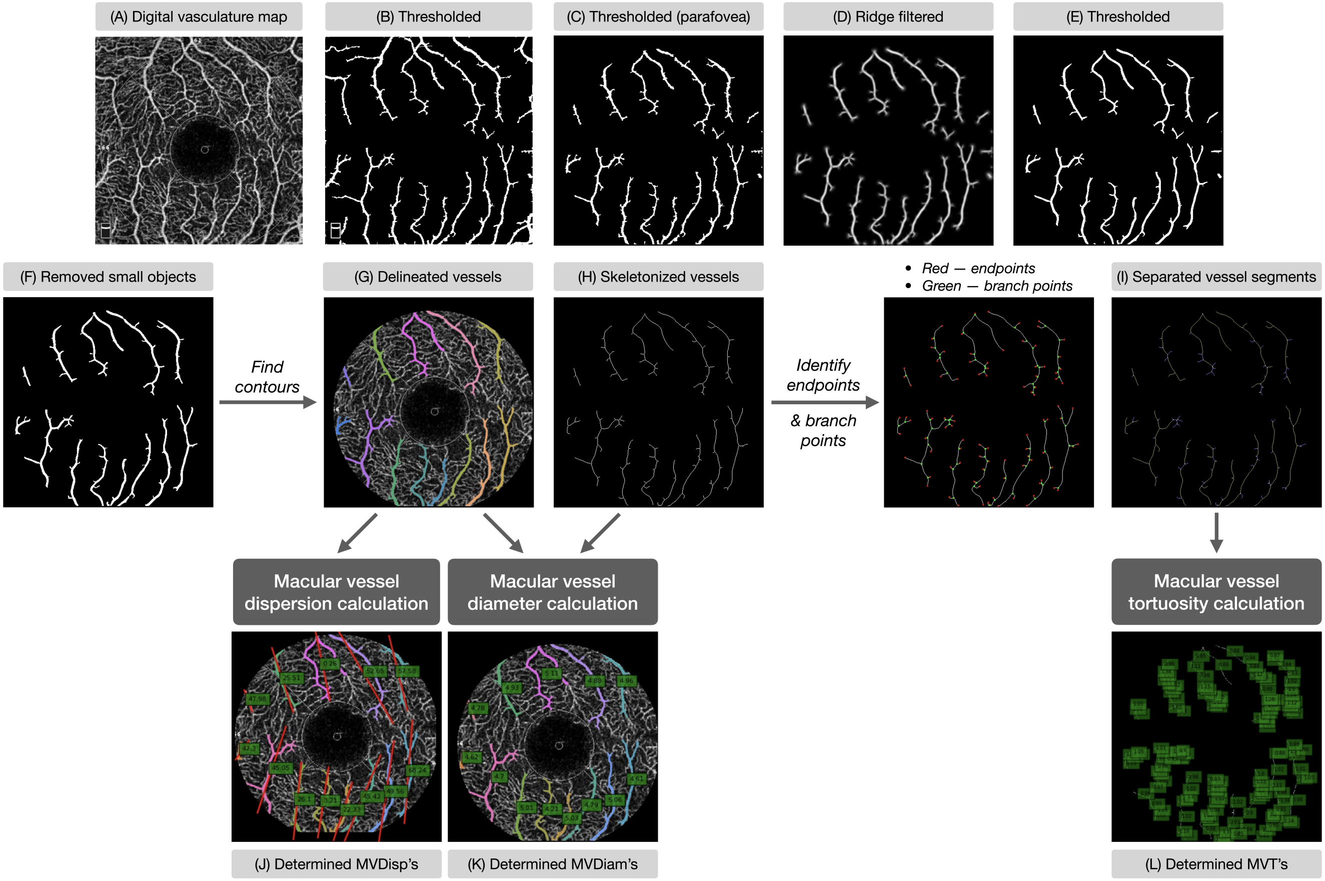

Figure 5: Process of retinal large vessel delineation and vessel segments separation.

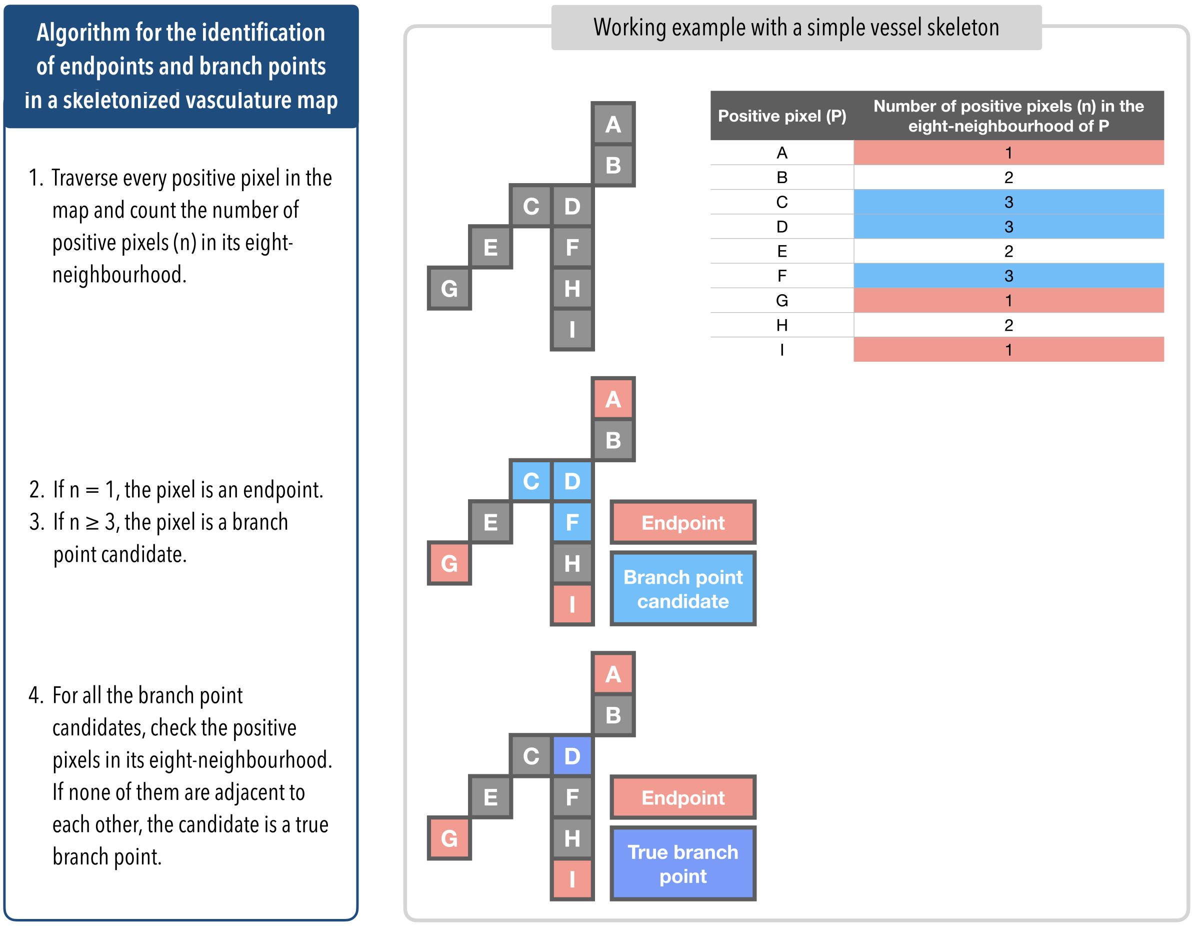

Positive pixel (P)	Number of positive pixels (n) in the eight-neighbourhood of P
A	1
B	2
C	3
D	3
E	2
F	3
G	1
H	2
I	1

Figure 6: Detection of endpoints and branch points in a skeletonized image.

2.3 Calculation of computational retinal microvascular biomarkers

2.3.1 Retinal capillaries biomarkers

Capillary perfusion density

Capillary perfusion density (PDC) was calculated for both DCPs and SCPs based on the generated DVM and the ETDRS grid accompanying it. The map was first converted to grayscale with pixel intensity values ranging from 0 (black) to 255 (white). Next, PDC was calculated as the average intensity of all the non-large-vessel pixels in the considered section of the map. Since brighter pixels (with larger intensity values) often correspond to vascular structures in OCTA images, when a map has a greater calculated PDC, it has denser capillaries.

Fractal dimension

Fractal dimension (FD) was used to assess the degree of complexity of the retinal microvascular structure in both DCPs and SCPs. In our study, we adaptively thresholded the extracted DVM and calculated its FD by applying the box-counting algorithm to the resulting binarized map [7].

2.3.2 Retinal large vessels biomarkers

Large vessels perfusion density

Large vessels perfusion density (PDL) was calculated based on the generated DVM in the SCP and its ETDRS grid. For a considered section of the map, PDL was calculated as the proportion of large-vessel pixels in it. When a map has a greater calculated PDL, it has denser large vessels.

Macular vessel dispersion

Macular vessel dispersion (MVDisp) was determined based on Figure 5G through the process illustrated in Figure 7. In a healthy eye, the parafoveal vessels are more centripetal towards the center of the fovea. MVDisp refers to the degree of centripetalism of the parafoveal vessels – the larger MVDisp is, the less centripetal the vessels are on average.

To determine MVDisp, Figure 5F was first padded and cropped so that the parafovea region was centered. Next, for each delineated retinal vessel V_i ($i = 1, \ldots, N$, where N is the total number of delineated vessels), the ellipse E_i with the same second moments as the region V_i covered was determined. If V_i was perfectly centripetal, it would be identically orientated as the line segment connecting the centroid

of E_i and the center of the fovea. MVDisp was thus defined to be the mean of all the angles between the major axis of E_i and the line segment L_i connecting the centroid of E_i and the center of C_{inner}, which corresponded to the fovea, identified when applying the ETDRS grid to the DVM.

$$\text{MVDisp} = \frac{1}{N}\sum_{i=1}^{N} \text{Angle between } L_i \text{ and the major axis of } V_i$$

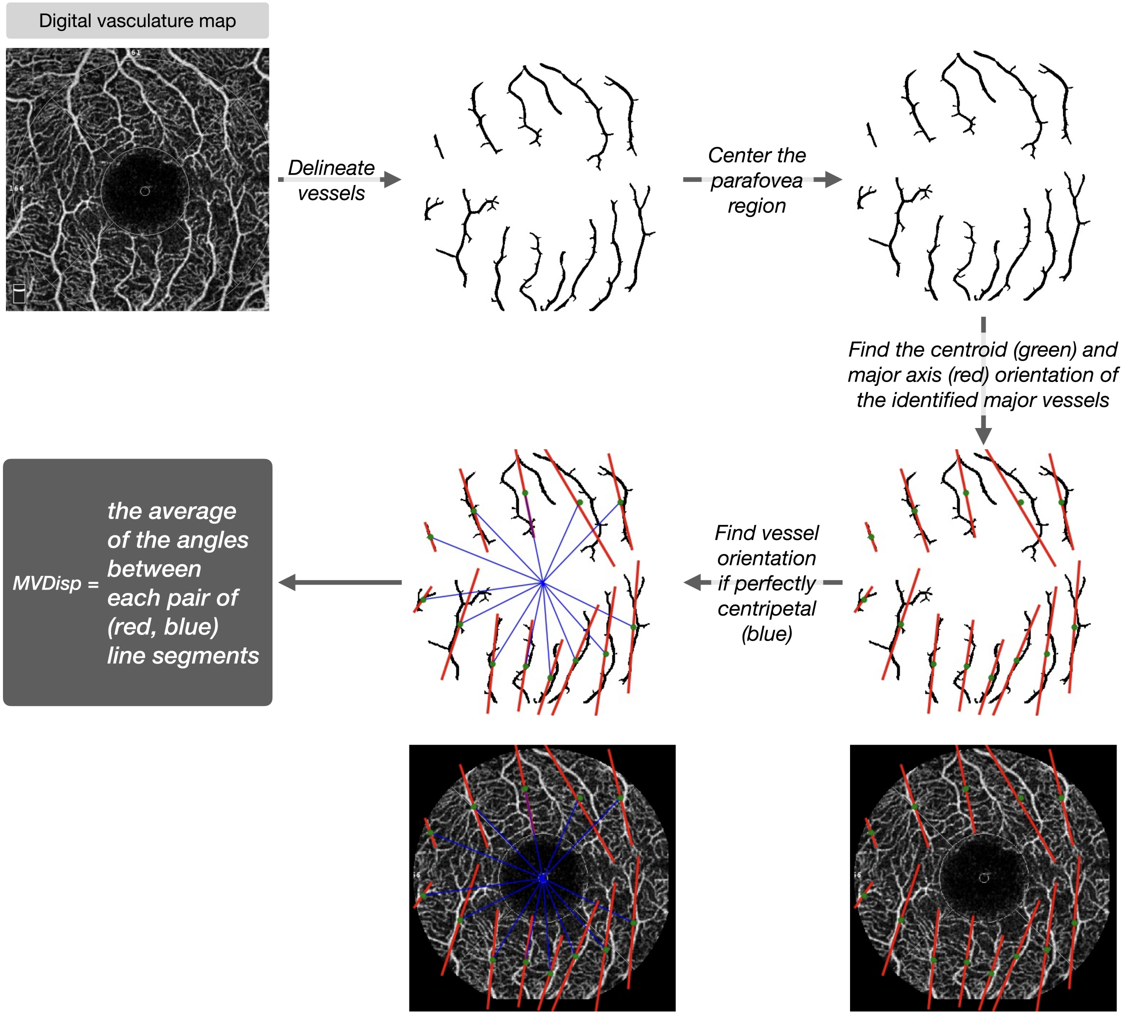

Figure 7: Calculation of MVDisp. Abbreviation used – MVDisp: macular vessel dispersion.

Macular vessel diameter

Macular vessel diameter (MVDiam) was determined using Figure 5G and Figure 5H. MVDiam was defined to be the mean of the ratios between the pixel area and the geodesic length of all the delineated large retinal vessels. Denoting S as the side length of the DVM, N as the number of delineated vessels and V_i as the ith delineated vessel in the image, the formula for MVDiam is:

$$\text{MVDiam} = \frac{1}{S \cdot N}\sum_{i=1}^{N} \frac{\text{Pixel area of } V_i}{\text{Geodesic length of } V_i}$$

Macular vessel tortuosity

Macular vessel tortuosity (MVT) describes how curved and twisty the path followed by each vessel is [8] and was calculated using Figure 5I. MVT was quantified as the mean of the ratios between the geodesic length and Euclidean length of the separated vessel segments [9]. Denoting M as the number of vessel segments and S_i as the ith vessel segment, the formula for MVT is:

$$\text{MVT} = \frac{1}{M}\sum_{i=1}^{M}\frac{\text{Geodesic length of } S_i}{\text{Euclidean length of } S_i}$$

2.3.3 Biomarkers related to the foveal avascular zone

Foveal avascular zone area

The FAZ area (FA) was calculated for both SCPs and DCPs. In clockwise order, denote the vertices of the demarcated FAZ polygon as $(a_1, b_1), (a_2, b_2), \ldots, (a_n, b_n)$. The area of this polygon can then be calculated by the shoelace formula:

$$\frac{1}{2}|(a_1b_2 + a_2b_3 + \ldots + a_nb_1) - (b_1a_2 + b_2a_3 + \ldots + b_na_1)|$$

FA is next defined to be the FAZ polygon area divided by the pixel area of the DVM. The standardization ensured that the FA is comparable across images of different sizes if the DVM is always focused on a 3×3 region. The correlation between Optovue's calculated FAZ area and the average of the superficial and deep FAZ area quantified in our implementation was 0.81, which was significantly different from 0 with a p-value < 0.001.

Foveal avascular zone acircularity

FAZ acircularity (FAC) quantifies the irregularity of the shape of the demarcated FAZ [10] and was calculated by the following formula over SCPs and DCPs:

$$\text{FACI} = \frac{\text{Perimeter of the FAZ polygon}}{\text{Circumference of the circle with an area equal to the FAZ polygon}}$$

When FAC is equal to one, the FAZ is a perfect circle. Greater FAC values indicate that the FAZ has more irregular shapes.

References

1. Seknazi, D. *et al.* Optical coherence tomography angiography in retinal vein occlusion: correlations between macular vascular density, visual acuity, and peripheral nonperfusion area on fluorescein angiography. *Retina (Philadelphia, Pa.)* **38,** 1562 (2018).

2. Otsu, N. A threshold selection method from gray-level histograms. *IEEE transactions on systems, man, and cybernetics* **9,** 62–66 (1979).

3. Hough, P. V. *Method and means for recognizing complex patterns* US Patent 3,069,654. Dec. 1962.

4. Harris, C. G., Stephens, M., *et al. A combined corner and edge detector.* in *Alvey vision conference* **15** (1988), 10–5244.

5. Pointer, M. A comparison of the CIE 1976 colour spaces. *Color Research & Application* **6,** 108–118 (1981).

6. Sato, Y. *et al. 3D multi-scale line filter for segmentation and visualization of curvilinear structures in medical images* in *CVRMed-MRCAS'97* (1997), 213–222.

7. Lemmens, S. *et al.* Systematic Review on Fractal Dimension of the Retinal Vasculature in Neurodegeneration and Stroke: Assessment of a Potential Biomarker. *Frontiers in neuroscience* **14,** 16 (2020).

8. Aghamohamadian-Sharbaf, M., Pourreza, H. R. & Banaee, T. A novel curvature-based algorithm for automatic grading of retinal blood vessel tortuosity. *IEEE Journal Of Biomedical and health informatics* **20,** 586–595 (2015).

9. Arrigo, A. *et al.* Vascular patterns in retinitis pigmentosa on swept-source optical coherence tomography angiography. *Journal of clinical medicine* **8,** 1425 (2019).

10. Conrath, J. *et al.* Semi-automated detection of the foveal avascular zone in fluorescein angiograms in diabetes mellitus. *Clinical & experimental ophthalmology* **34,** 119–123 (2006).
